# Hox Proteins Display a Common and Ancestral Ability to Diversify Their Interaction Mode with the PBC Class Cofactors

**DOI:** 10.1371/journal.pbio.1001351

**Published:** 2012-06-26

**Authors:** Bruno Hudry, Sophie Remacle, Marie-Claire Delfini, René Rezsohazy, Yacine Graba, Samir Merabet

**Affiliations:** 1 Institut de Biologie du Développement de Marseille Luminy, IBDML, UMR7288, CNRS, AMU, Parc Scientifique de Luminy, Case 907, Marseille, France; 2 Molecular and Cellular Animal Embryology Group, Life Sciences Institute, Université catholique de Louvain, Louvain-la-Neuve, Belgium; University of Cambridge, United Kingdom

## Abstract

Hox transcription factors control a number of developmental processes with the help of the PBC class proteins. In vitro analyses have established that the formation of Hox/PBC complexes relies on a short conserved Hox protein motif called the hexapeptide (HX). This paradigm is at the basis of the vast majority of experimental approaches dedicated to the study of Hox protein function. Here we questioned the unique and general use of the HX for PBC recruitment by using the Bimolecular Fluorescence Complementation (BiFC) assay. This method allows analyzing Hox-PBC interactions in vivo and at a genome-wide scale. We found that the HX is dispensable for PBC recruitment in the majority of investigated *Drosophila* and mouse Hox proteins. We showed that HX-independent interaction modes are uncovered by the presence of Meis class cofactors, a property which was also observed with Hox proteins of the cnidarian sea anemone *Nematostella vectensis*. Finally, we revealed that paralog-specific motifs convey major PBC-recruiting functions in *Drosophila* Hox proteins. Altogether, our results highlight that flexibility in Hox-PBC interactions is an ancestral and evolutionary conserved character, which has strong implications for the understanding of Hox protein functions during normal development and pathologic processes.

## Abstract 2

Hox protein function during development and evolution relies on conserved multiple interaction modes with cofactors of the PBC and Meis families.

## Author Summary

Hox proteins are key transcriptional regulators of animal development, famously helping to determine identity along the anterior-posterior body axis. Although their evolution and developmental roles are well established, the molecular mechanisms underlying their specific functions remain poorly characterized. The current dominant view is that interaction with different members of the PBC family of transcription factors confers specific DNA-binding properties on different Hox proteins. However, this idea conflicts with in vitro evidence that a short “hexapeptide” (HX) motif shared by most Hox proteins is solely responsible for generic PBC recruitment. Here we have used the BiFC (bimolecular fluorescence complementation) method to address the global importance of the HX motif for Hox-PBC interactions in living cells and living animals including fruit flies and chick embryos. We observe that most interactions between Hox and PBC proteins do not depend on HX, and that alternative protein motifs are widely used for PBC recruitment in vivo. We also show that DNA binding by a second family of cofactors, the Meis proteins, unmasks these alternative interaction modes and that this property is conserved not only across Bilateria, but also in the basal animal phylum Cnidaria. Taken together, our results demonstrate that Hox-PBC partnership relies on multiple interaction modes, which can be influenced by additional transcriptional partners. We propose that this ancestral feature has been essential for ensuring Hox functional plasticity during development and evolution.

## Introduction

Hox genes encode homeodomain (HD)-containing transcription factors that specify cell fates along the anterior-posterior (AP) axis of all bilaterian embryos [1]. Besides early patterning functions, Hox genes are involved in the morphogenesis of various organs [2] and in the homeostasis of different cell types in adults [3],[4]. Hox functional diversity is accompanied by a high level of transcriptional specificity, as illustrated by the distinct developing programs triggered by each Hox protein during embryogenesis. These specific functions contrast with the poor DNA-binding stringency of Hox HDs, which fall only into two specificity groups [5],[6].

Hox DNA-binding specificity is enhanced by interactions with two families of cofactors, being collectively referred to as PBC and Meis [7]. These cofactors belong to the TALE (three amino acids loop extension) class of HD-containing transcription factors. Representatives of PBC are the *Drosophila* Extradenticle (Exd) or vertebrate Pbx1–4 proteins. The Meis family comprises Meis and Prep subclasses in vertebrates, but *Drosophila* has only one Meis representative called *homothorax* (*hth*) [7]. Meis family members are required for the nuclear translocation of PBC proteins, through interactions via a highly conserved domain localized in the N-terminal part of both partners [8]. The role of PBC proteins as direct Hox cofactors is well established [9], allowing the formation of Hox/PBC/Meis complexes regulating several well-characterized target genes [10] in different developmental contexts [11].

PBC proteins form cooperative DNA-binding complexes with Hox proteins from paralog groups 1 to 10 [12]. The formation of Hox/PBC complexes not only improves Hox DNA-binding affinity but also extends the size of cognate DNA sequences. Moreover, it was shown that the identity of the two central nucleotides in the Hox-PBC binding site could discriminate the Hox protein engaged in the Hox/PBC complex [13], although this observation does not apply to all characterized Hox target enhancers [14]. Recent studies further established that the interaction with the PBC cofactor helps *Drosophila* Hox proteins to recognize distinct DNA-structures [15],[16]. This recognition mode was shown to rely on conserved and paralog-specific residues [15], and it was suggested that the PBC cofactor could be widely used to unlock a “latent specificity” in Hox DNA-binding recognition properties [16].

Biochemical and structural analyses of Hox/PBC complexes have identified a generic mode of interaction whereby the recruitment of PBC cofactors is dependent on a six-residue-long peptide lying upstream of the Hox HD. This hexapeptide (HX) motif contains a core Y/FP/DWM sequence in Hox paralog groups 1–8, and is considerably divergent in posterior Hox paralog groups 9–10 that retain only a single conserved W residue. This residue establishes crucial contacts within a hydrophobic pocket formed in part by the TALE motif of the PBC HD [15],[17]–[19]. Accordingly, the mutation of the W residue is often sufficient to abolish Hox cooperative DNA binding with PBC proteins in vitro [20],[21]. However, in the case of vertebrate proteins, data regarding Hox-PBC interactions were mostly obtained with nearly identical Hox/PBC binding sites initially derived from a sequence (called *PRS* for Pbx Recognition Sequence) requiring the HX for Hox/PBC complex assembly [20]–[25]. These binding sites thus presented a strong bias towards HX-dependent association modes. Finally, several Hox/PBC structures have been solved with vertebrate and invertebrate proteins (see [10] for review). These structures were obtained using Hox peptides limited to the region encompassing the HD and HX, which excludes protein domains that could additionally contribute to the interaction with PBC. Such protein domains have been described in the C-terminal part of the *Drosophila* Deformed (Dfd, [26]) and Ultrabithorax (Ubx, [27]) proteins, and it was later found that a short motif lying immediately downstream of the HD could convey Exd-recruiting activities in Ubx [28]. Nevertheless, the general requirement of the HX for Hox-PBC interactions in vitro has led to the assumption that this motif is the main, if not unique, Hox protein motif used for Hox/PBC complex assembly in vivo.

The proposal of a single generic HX-mediated interaction mode needs to be reconsidered in light of several in vivo phenotypes associated with the HX mutation. In the mouse Hoxb6 [29], Hoxb8 [30], or Hoxa9 [31], the HX mutation does not systematically abolish transforming activities expected to be PBC-dependent in hematopoietic cells. In addition, the lack of the HX in mouse Hoxb8 leads to dominant-negative phenotypes that are difficult to reconcile with PBC-dependent functions [32]. Finally, the HX mutation renders a truncated Labial (Lab) protein hyperactive in an Exd-dependent context [33], and does not compromise the Exd-dependent repression of the *Distalless* (*Dll*) enhancer by Ubx [34] and AbdominalA (AbdA, [35]) in the *Drosophila* embryo. Whether these observations constitute peculiar cases or whether Hox proteins frequently display HX dispensability for mediating interactions with the PBC cofactor remain, however, to be determined.

In this work, we investigated the requirement of the HX in vertebrate and invertebrate Hox proteins, focusing on several Hox paralog groups. Our analysis relied on the Bimolecular Fluorescence Complementation (BiFC) technology [36] to visualize Hox-PBC interactions in the *Drosophila* embryo, mammalian cells, and chick embryos. This method allows assessing the requirement of the HX in vivo. Importantly, BiFC presents the advantage of providing, for the first time, a global measure of protein domain requirement for Hox-PBC interactions. Indeed, a loss of fluorescent signal following protein domain mutation will reflect a broad use of the domain for the interaction, demonstrating its requirement in the regulation of a large set of target genes. Results showed a large dispensability of the HX for Hox-PBC interactions and identify widely used alternative interaction modes. We also demonstrated that the HX dispensability is often unmasked by the additional DNA binding of Meis proteins, and that both dispensability and Meis unmasking constitute an ancestral character of Hox proteins.

## Results

### The HX Is Dispensable in Several *Drosophila* Hox Proteins for PBC Recruitment In Vivo

**Figure 1:**
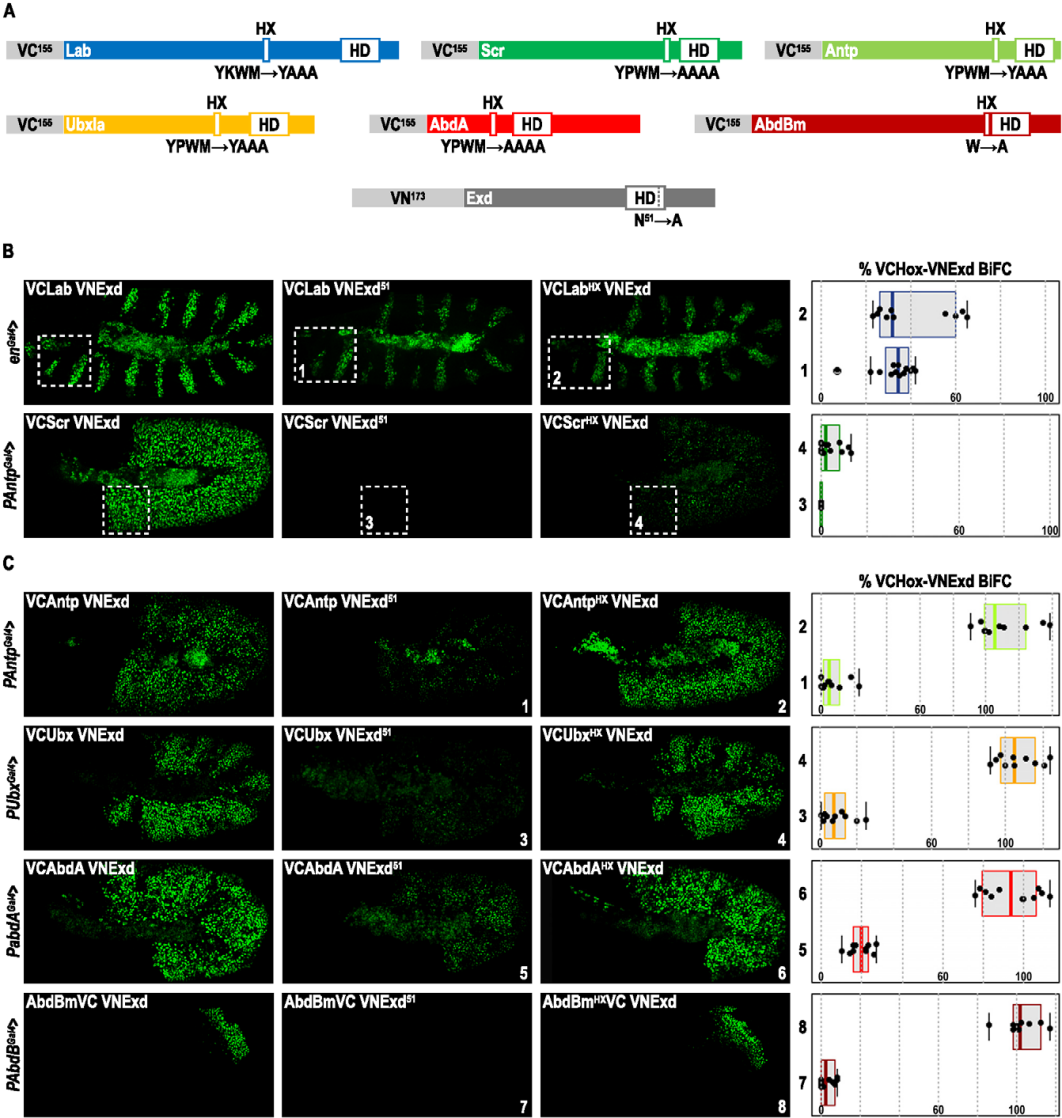
BiFC analysis of the HX requirement for Hox-Exd interactions in the *Drosophila* embryo. (A) Scheme of Hox and Exd fusion proteins used as UAS constructs for BiFC analysis in the *Drosophila* embryo. The place of the HX with regard to the Homeodomain (HD) is to scale for each Hox protein. The HX mutation engineered in each Hox protein is indicated, as well as the HD mutation in Exd. VC and VN correspond to the C-terminal and N-terminal fragments of the Venus fluorescent protein, respectively. (B–C) Images are illustrative confocal captures of stage 10 living embryos. Each lane corresponds to a different Hox protein, whose fusion variants are expressed with a specific Gal4 driver, as indicated on the left. First and second columns correspond to BiFC between Hox and the wild type or HD-mutated form of Exd, respectively. The third column corresponds to BiFC between Exd and the HX-mutated Hox proteins. Panels on the right show the statistical quantification, as a boxplot representation, of fluorescent signals resulting from BiFC in each condition (see also Materials and Methods). Quantifications with mutated Exd and Hox proteins are numbered and are represented as a percentage of the BiFC normally obtained with the corresponding wild type proteins. Dotted-white boxes in (B) indicate the zone where BiFC signals have been quantified. See also Figures S1, S2, S3, S4.

BiFC relies on the property of N- and C-terminal fragments of fluorescent proteins to reconstitute fluorescence once they are brought in close proximity. This property was used in different model systems to validate the existence of direct interactions between two proteins, each fused to a non-fluorescent N- or C-terminal fragment [36]. Here we have investigated the global contribution of the HX of *Drosophila* Hox proteins for Exd recruitment by comparing fluorescent signals resulting from the assembly of Exd with wild type or HX-mutated Hox proteins. Six of the eight *Drosophila* Hox proteins were fused to the C-terminal part (VC) of Venus (a variant of the Green Fluorescent Protein) either as wild type or HX-mutated versions (Figure 1A and Table S1). The complementary N-terminal part of Venus (VN) was fused to the Exd cofactor. This choice of fusion topologies was based on previous results using AbdA and Exd for establishing BiFC in the *Drosophila* embryo [37]. In particular, it was shown that the combination with VC-AbdA and VN-Exd was best suited for BiFC since these fusion topologies did not affect known regulatory functions in vivo [37]. Constructs were cloned downstream of Gal4 UAS sequences for expression through the UAS/Gal4 system. Wild type and HX-mutated forms of Hox proteins were inserted at the same genomic locus ([38] and Materials and Methods), allowing similar levels of protein expression (Figure S1). For Antennapedia (Antp), Ubx, AbdA, and AbdominalB (AbdB) fusion proteins, we used Gal4 drivers derived from P insertions in the corresponding genes, which reproduce the endogenous Hox gene expression profile [37],[39]. These genetic tools allow measuring BiFC in cells that normally express the Hox and Exd proteins during embryonic development. In addition, the *P(Ubx-Gal4)*, *P(abdA-Gal4)*, and *P(AbdB-Gal4)* correspond to null mutations, allowing expressing fusion proteins in the absence of the endogenous Hox product [37],[39]. Under our experimental parameters, fusion proteins were expressed at levels close to physiological conditions and BiFC was analyzed in the context of a perfectly viable embryo (as previously described for AbdA [37] and Figure S1). Since no P insertions are available in close proximity of *labial* (*lab*) and *Sex combs reduced* (*Scr*), we used the *engrailed (en)-Gal4* and *Antp-Gal4* drivers to express the Lab and Scr fusion proteins, respectively. In these contexts, BiFC was analyzed in segments normally specified by Lab and Scr, with levels of fusion protein not affecting embryonic development (Figure S1).

We observed that all VC-Hox/VN-Exd complexes produce fluorescent signals in the epidermis of the *Drosophila* embryo (Figure 1B–C). By comparison, no BiFC signals were visualized between VC-Hox proteins and the transcription factor Collier (Col) fused to the VN fragment (Figure S2). The specificity of BiFC in the *Drosophila* embryo was also previously verified by several control experiments [37]. Among these, it was shown that using AbdA and Exd fusion proteins mutated in the residue 51 of the HD abolished DNA binding and complex formation in vitro as well as BiFC in vivo [37]. Since the other *Drosophila* Hox proteins also require the DNA binding of Exd for dimeric complex formation in vitro (Figure S3), we performed BiFC between wild type VC-Hox fusion proteins and the HD-mutated form of VN-Exd. We observed that abolishing complex formation in vitro correlates with a strong decrease of BiFC, although to a lesser extent for Lab (Figure 1B–C). This latter result suggests that a fraction of Lab-Exd interactions could occur outside the DNA in vivo. Altogether, these control experiments highlight that Hox/Exd complex assembly depends, for a large part, on interactions occurring on DNA. They also demonstrate that BiFC properly reproduces conditions affecting Exd recruitment for all *Drosophila* Hox proteins used in this study.

We next investigated the contribution of the HX for Hox-Exd interactions, by using HX-mutated forms of Hox proteins for BiFC analysis. Lab and Sex combs reduced (Scr) show a clear dependency for the HX: Lab loses around 70% of the BiFC signal, whereas for Scr the HX mutation almost completely abolishes BiFC (Figure 1B). In both cases, the loss was similar to the HD mutation of Exd, suggesting that all Lab/Exd or Scr/Exd complexes occurring on DNA were affected. For Antp, Ubx, AbdA, and AbdB, results show that BiFC is not significantly affected upon the HX mutation (Figure 1C). In addition, BiFC with the HD-mutated form of Exd further establishes that Hox-Exd interactions are still mainly occurring on DNA in the context of the HX mutation (Figure S4).

We conclude that all but Lab and Scr *Drosophila* Hox proteins marginally require the HX for Exd interaction in vivo.

### The HX Is Dispensable in Several Mouse Hox Proteins for PBC Recruitment In Vivo

**Figure 2:**
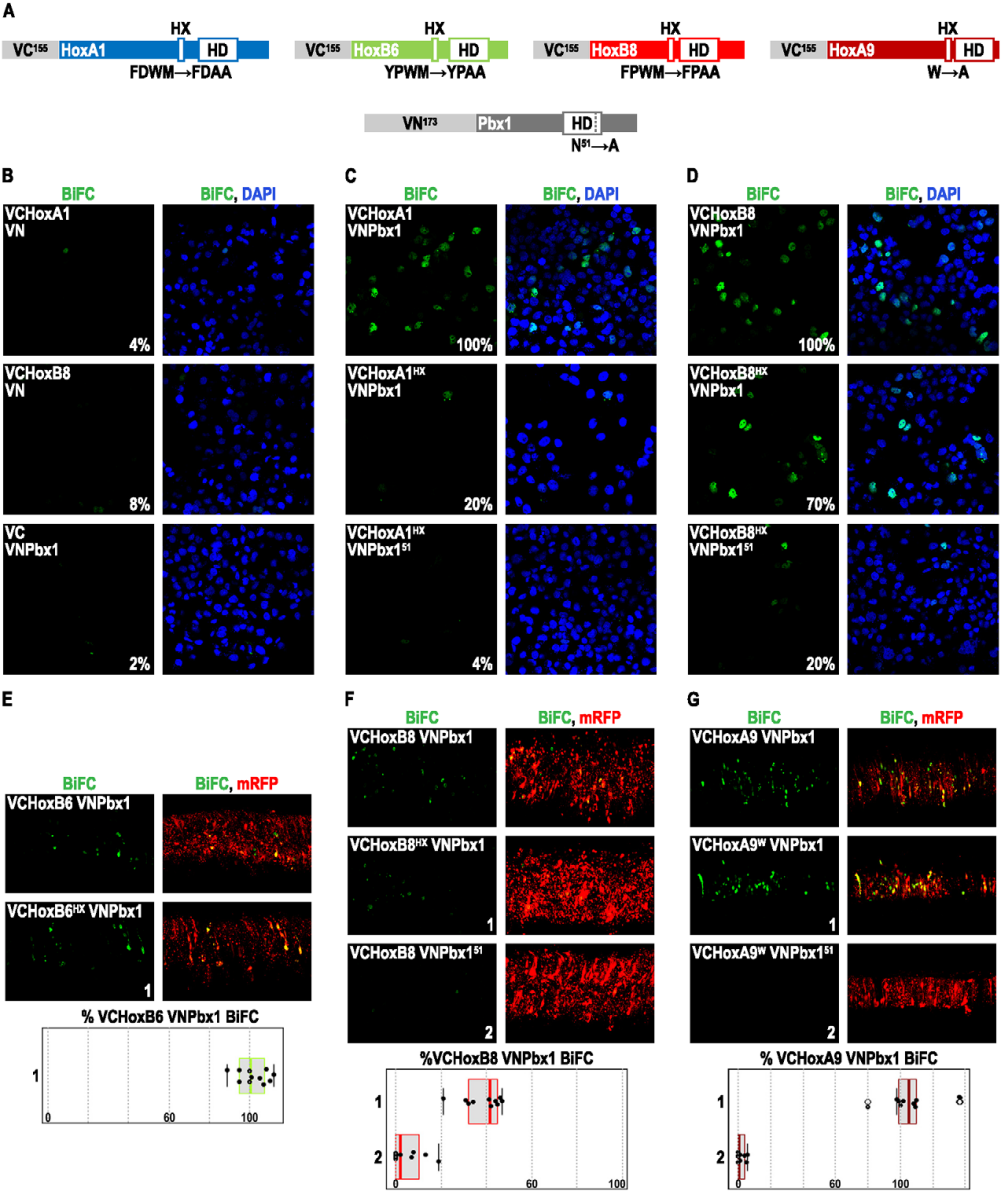
BiFC analysis of the HX requirement for Hox-Pbx1 interactions in mammalian COS7 cells and the trunk neural tube of the chick embryo. (A) Scheme of Hox and Pbx1 fusion proteins used for BiFC analysis. The HX mutation engineered in each Hox protein is indicated, as well as the HD mutation in Pbx1. (B–D) BiFC between Hox and Pbx1 fusion proteins in mammalian COS7 cells. (B) BiFC between Hoxa1, Hoxb8, or Pbx1 fusion proteins and the complementary isolated VN or VC fragment, as indicated. (C) BiFC between Hoxa1 and Pbx1 fusion proteins. (D) BiFC between Hoxb8 and Pbx1 fusion proteins. Wild type and mutated fusion constructs used in each transfection experiment are indicated. Illustrative confocal pictures of BiFC signals (green) in COS7 cells are shown with (first column) or without (second column) nuclei staining (with DAPI, blue). The percentage of BiFC levels was deduced from the quantification of the intensity and number of fluorescent signals in approximately 300 cells (Materials and Methods). Experiments were independently repeated three times. See also Figure S5. (E–G) BiFC between Hox and Pbx1 fusion proteins in the trunk neural tube of the chick embryo. (E) BiFC between Hoxb6 and Pbx1 fusion proteins. (F) BiFC between Hoxb8 and Pbx1 fusion proteins. (G) BiFC between Hoxa9 and Pbx1 fusion proteins. Wild type and mutated fusion constructs used in each experiment are indicated. Fusion proteins were co-electroporated with a mRFP-encoding vector (red) for assessing the efficiency of the electroporation (Materials and Methods). The quantification of BiFC signals (green) in one hemisegment is presented as boxplots in graphs on the bottom. Quantifications with mutated fusion proteins are numbered and represented as a percentage of BiFC normally obtained with the corresponding wild type fusion proteins. See also Figure S5.

The global contribution of the HX of vertebrate Hox proteins for Pbx1 recruitment in vivo was also assessed by BiFC. Selected mouse Hox proteins representative of anterior, central, and posterior classes and Pbx1 proteins were respectively fused to the C-terminal and N-terminal fragments of Venus (Figure 2A). All constructs were cloned downstream the pCMV promoter for expression (Materials and Methods and Table S1).

We first performed BiFC using Pbx1, Hoxa1, and Hoxb8 proteins in mammalian COS7 cells. Of note, these cells endogenously express Meis1 [40], and Hoxa1 and Hoxb8 are the only vertebrate Hox proteins for which the functional role of the HX has been examined in vivo [32],[41].

The specificity of BiFC in these experiments was established by the absence of signals between VC-Hox fusion proteins and the Venus VN fragment, and between the VN-Pbx1 fusion protein and the Venus VC fragment (Figure 2B and Figure S5 for expression level controls).

We observed that VC-Hoxa1 and VC-Hoxb8 proteins produce BiFC signals with VN-Pbx1 in COS7 cells (Figure 2C–D). As for *Drosophila* Hox/Exd complexes, loss of BiFC with a HD-mutated form of Pbx1 demonstrates that Hoxa1/Pbx1 and Hoxb8/Pbx1 interactions mainly occur on DNA (Figure 2C–D). The role of the HX was then analyzed by using HX-mutated forms of Hoxa1 and Hoxb8. We found that this mutation has distinct consequences in each protein, with a mean loss of BiFC of 80% for HX-mutated Hoxa1 and 30% for HX-mutated Hoxb8 (Figure 2C–D). Thus, Hoxa1-Pbx1 interactions are strongly dependent on the HX, while most of Hoxb8-Pbx1 interactions do not require this motif in COS7 cells.

We next investigated the PBC-recruiting function of the HX of vertebrate Hox proteins in the chick embryo, extending our analyses to Hoxb6 and Hoxa9. Heterologous gene expression of mouse Hox proteins was achieved following electroporation in the trunk neural tube, which endogenously expresses Hox [42] and PBC/Meis proteins [43]. Hox and Pbx1 fusion proteins were electroporated at identical E2 embryonic stages and BiFC was observed 24 h later (Materials and Methods). Three of the four investigated Hox proteins produce BiFC with Pbx1 (Figure 2E–G). Hoxa1 is the only protein that did not produce BiFC (unpublished data). This negative result has, however, to be taken cautiously, as resident central Hox proteins may, according to the phenomenon of posterior prevalence, suppress the activity of the heterologously expressed Hoxa1 protein. In this case, posterior prevalence could partly rely on competition with the PBC cofactor, as recently suggested [44]. Nonetheless, this negative result indicates that the trunk neural tube is not appropriate for revealing interactions between Pbx1 and Hox proteins of anterior paralog groups.

The role of the HX was thus analyzed for the central and posterior Hoxb6, Hoxb8, and Hoxa9 proteins. We found that the HX mutation does not affect Hoxb6-Pbx1 (Figure 2E) and Hoxa9-Pbx1 (Figure 2G) interactions, while it leads to mean loss of 60% of BiFC between Hoxb8 and Pbx1 (Figure 2F). The latter result is consistent with BiFC in COS7 cells, which also established a significant contribution of the HX for the interaction with Pbx1. Specificity of BiFC in the chick embryo was confirmed by the absence of signal when the HD-mutated form of Pbx1, which properly localizes in nuclei of neural cells (see Figure S5 for a comparison with the wild type Pbx1 fusion protein), was used (Figure 2F–G).

These data, together with those in the *Drosophila* embryo, demonstrate that Hox proteins display an unexpected level of HX-dispensability for interacting with PBC-class proteins in vivo.

### The HX Is Dispensable in Several *Drosophila* Hox Proteins for PBC Recruitment In Vitro

To further investigate the contribution of the HX for Hox-Exd interactions, we performed electrophoretic mobility shift assays (EMSAs) on two types of DNA probes. The first type, represented by *Dllcon*, is derived from the *Dll* enhancer [45] and has the property to allow the formation of Hox/Exd and/or Hox/Exd/Hth complexes with all *Drosophila* Hox proteins. The second type corresponds to DNA-binding sites characterized from natural *cis*-regulatory sequences of Hox target genes. The contribution of the HX was evaluated in the context of Hox/Exd (when applicable) and Hox/Exd/Hth complexes. The latter allows assessing the impact of Hth in Hox-Exd interactions.

**Figure 3:**
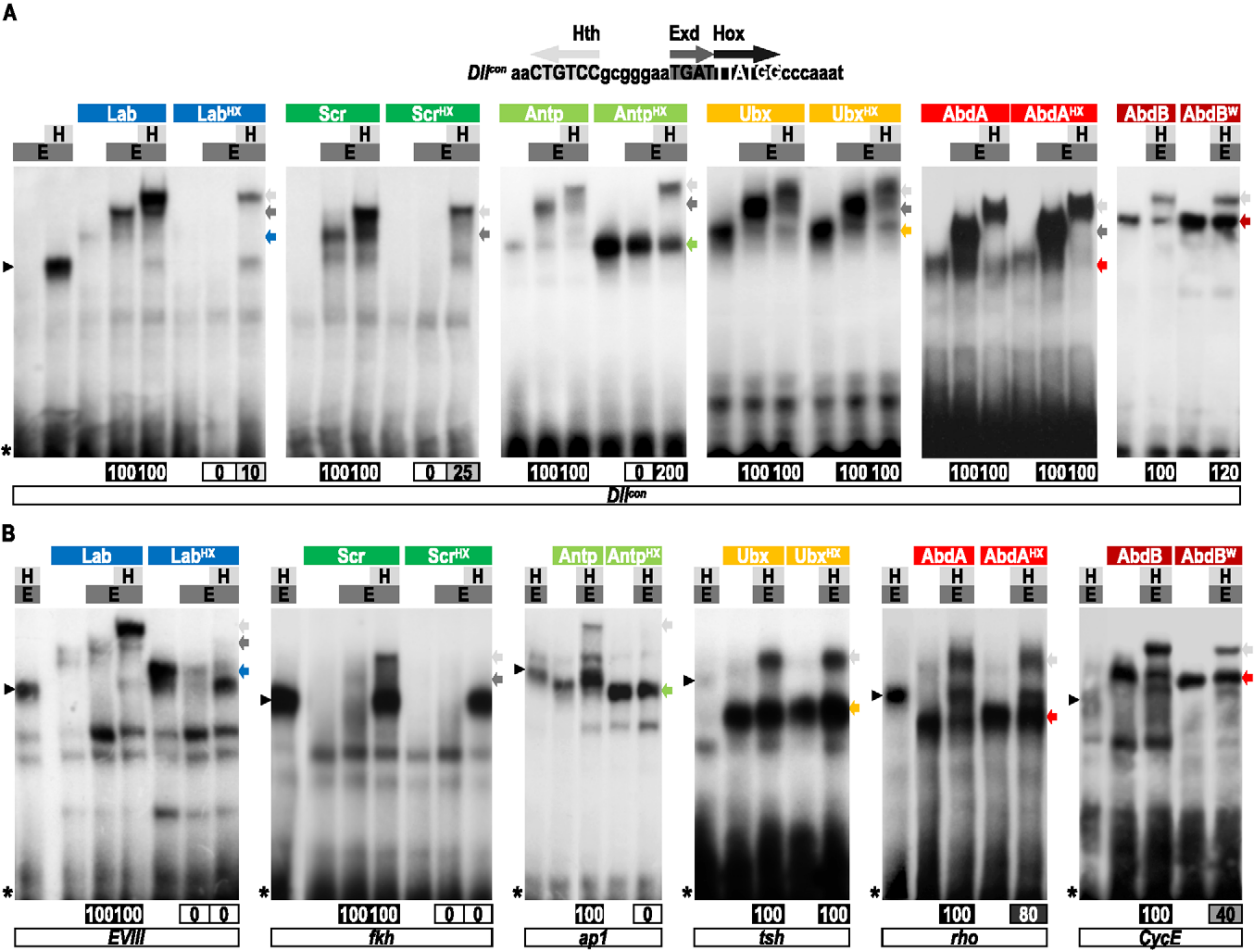
Role of the HX of *Drosophila* Hox proteins for Exd and/or Exd/Hth recruitment in vitro. (A) Electomobility shift assays (EMSAs) on the *Dllcon* probe. (B) EMSAs on physiological probes. In all panels, wild type and HX-mutated Hox proteins are indicated above each gel (colored bars, with a same color code as in Figure 1A). The presence of Exd (E) and Hth (H) cofactors is schematized by dark and light gray bars, respectively. On the right of each gel, colored arrows indicate the corresponding Hox monomer binding. Gray and black arrows indicate Hox/Exd and Hox/Exd/Hth complexes, respectively. On the left, black arrowheads and asterisks depict Exd/Hth complexes and radioactive probes, respectively. Bands corresponding to dimeric and trimeric complexes have been quantified and are symbolized by gradient gray boxes below each gel. For each Hox protein, the effect of the HX mutation is numbered as a percentage of remaining complexes when compared to the wild type Hox protein. Name of physiological probes (the nucleotides sequences are provided in Materials and Methods): fragment *EVIII* is derived from the enhancer of the Lab target gene *CG11339* [14]; *fkh* is derived from the enhancer of the Scr target gene *forkhead* [66]; *ap1* is derived from the enhancer of the Antp target gene *apterous* [67]; *tsh* is derived from the enhancer of the Ubx target gene *teashirt* [68]; *rho* is derived from the enhancer of the AbdA target gene *rhomboid* [58]; and *cycE* is derived from the enhancer of the AbdA target gene *cyclinE* [46]. See also Figure S6.

On the *Dllcon* probe, all tested Hox proteins form dimeric and trimeric complexes with Exd or Exd and Hth (Figure 3A), with the exception of AbdB, which forms only trimeric AbdB/Exd/Hth complexes (Figure 3A and unpublished data). In the absence of Hth, the HX mutation leads to a complete loss of Hox/Exd complex formation for Lab, Scr, and Antp (Figure 3A). As previously described [34],[35], the Ubx/Exd and AbdA/Exd dimeric complexes are not affected upon the HX mutation (Figure 3A).

We next investigated the requirement of the HX for Hox/Exd complex assembly in the presence of Hth. In that context, the HX mutation does not impair the potential of Ubx and AbdA to interact with Exd (Figure 3A). Interestingly, the presence of Hth either completely (for Antp) or weakly (for Lab and Scr) rescues the loss of Hox/Exd dimeric complex formation induced by the HX mutation (Figure 3A). Finally, the HX mutation in AbdB also has no effect on the trimeric complex assembly (Figure 3A). For all except AbdB, no Hox/Hth complexes can be formed on *Dllcon*, either with wild type or HX-mutated forms of Hox proteins (Figure S6). We concluded that the rescue of the HX mutation in the context of the trimeric complex is likely indicative of changes in Hox-Exd interactions. In the case of AbdB, we observed the formation of dimeric complexes with Hth, but only with the HX-mutated form and in absence of Exd (Figure S6). This suggests that the HX mutation uncovers AbdB-Hth contacts that could potentially be involved in the formation of AbdB/Exd/Hth complexes.

EMSAs with probes designed from physiological DNA-binding sites showed that Lab (*EVIII* probe), Scr (*fkh* probe), and Antp (*ap1* probe) require the HX to recruit Exd in the presence of Hth (Figure 3B). Of note, the strong monomer binding of the HX-mutated form of Lab is titrated out in the presence of Exd (Figure 3B), suggesting that the two proteins interact in a DNA-independent manner, as observed in vivo by BiFC (Figure 1B). For Ubx, AbdA, and AbdB, only trimeric complexes are observed on their respective natural DNA-binding sites (Figure 3B). In AbdB (*CycE* probe), the HX mutation affects but does not abolish the trimeric complex formation (Figure 3B, [46]). Of note, no AbdB/Hth complexes can be observed in that context (Figure S6). In Ubx (*tsh* probe) and AbdA (*rho* probe) the HX mutation has no or little effects, respectively (Figure 3B).

In conclusion, EMSAs on consensus and physiological binding sites support the view that all except Lab and Scr *Drosophila* Hox proteins have the potential to interact with Exd in the absence of the HX. In addition, results with the consensus nucleotide probe highlight two modes of HX-dispensability: the first one can be independent of Hth (Ubx and AbdA), while the second requires the presence of this third partner (other Hox proteins).

### The HX Is Dispensable in Several Mouse Hox Proteins for PBC Recruitment In Vitro

**Figure 4:**
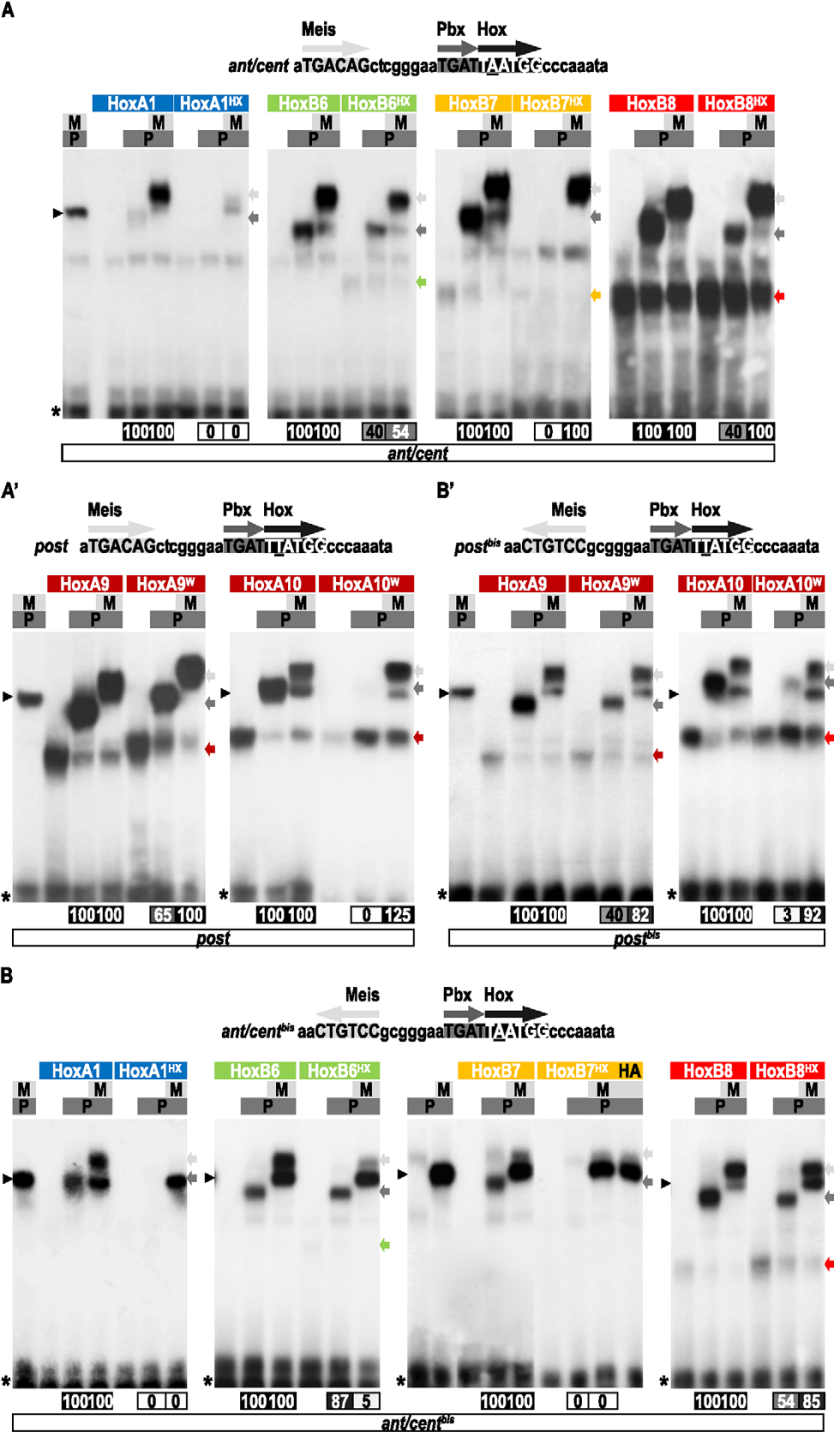
Role of the HX of mouse Hox proteins for Pbx1 and Pbx1/Meis1 recruitment in vitro. (A) EMSAs of wild type and HX mutated forms of Hoxa1, Hoxb6, Hoxb7, and Hoxb8 with the Pbx1 and Meis1 cofactors on the *ant/cent* probe, as indicated. (A′) EMSAs of wild type and HX mutated forms of Hoxa9 and Hoxa10 with the Pbx1 and Meis1 cofactors on the *post* probe, as indicated. (B) EMSAs of wild type and HX mutated forms of Hoxa1, Hoxb6, Hoxb7, and Hoxb8 with the Pbx1 and Meis1 cofactors on the *ant/cent^bis^* probe, as indicated. (B′) EMSAs of wild type and HX mutated forms of Hoxa9 and Hoxa10 with the Pbx1 and Meis1 cofactors on the *post^bis^* probe, as indicated. The sequence of each probe with orientations of the Hox, Pbx, and Meis binding sites are indicated above the gel. Colored bars, marks, and quantifications of protein complexes are symbolized as in Figure 3. See also Figures S7 and S8.

**Figure 5:**
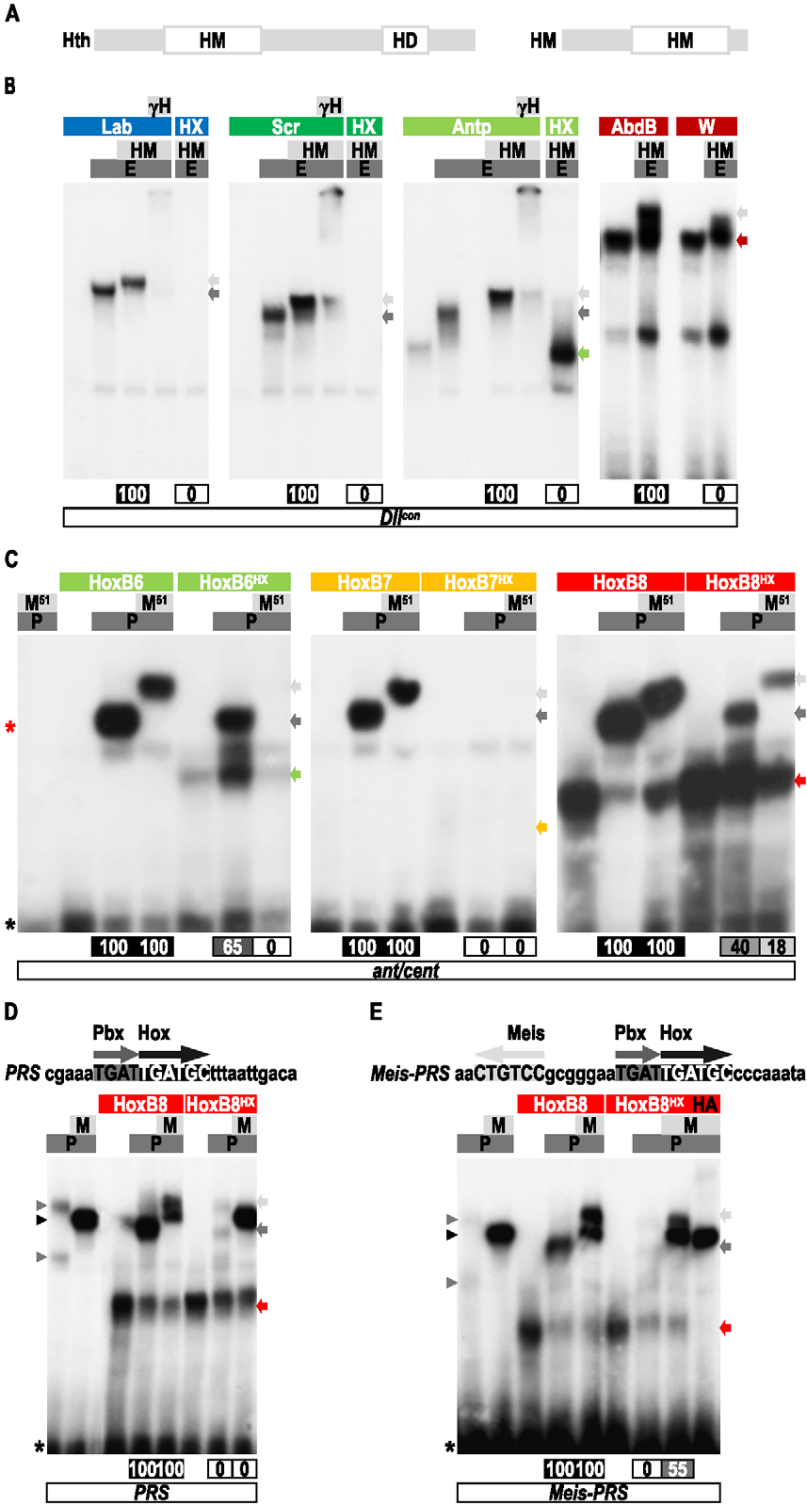
Meis class proteins cannot reveal HX-independent interaction modes in the absence of DNA-binding. (A) Scheme of the short HD-less isoform of Hth, which contains only the Exd interaction domain (HM). This form was fused to the HA tag (not schematized). (B) EMSAs with wild type or HX-mutated forms of *Drosophila* Hox proteins and Exd and the HD-less isoform of Hth on the *Dllcon* probe, as indicated. For Hox proteins making a dimeric complex with Exd (Lab, Scr and Antp), an anti-HA raised against Hth-HM was also used to validate the presence of the trimeric complex. In all cases, the HX mutation abolishes trimeric complex formation with Exd and Hth-HM. (C) EMSAs with wild type or HX-mutated forms of central mouse Hox proteins and Pbx1 and the HD-mutated form of Meis1a on the *ant/cent* nucleotide probe, as indicated. The HD mutation abolishes DNA-binding [48], as exemplified by the absence of DNA-bound Pbx/Meis complexes (red asterisk). This mutation also abolishes (for Hoxb6 and Hoxb7) or drastically affects (for Hoxb8) trimeric complex formation with HX-mutated Hox proteins. (D) EMSAs with wild type or HX-mutated forms of Hoxb8 and Pbx1 or Pbx1/Meis1 on the *PRS* nucleotide probe, as indicated. On this probe, the HX-mutated form of Hoxb8 is not able to form any dimeric or trimeric complex with Pbx1 or Pbx1/Meis1, respectively. (E) EMSAs with wild type or HX-mutated forms of Hoxb8 and Pbx1 or Pbx1/Meis1 on the *PRS* probe containing a Meis binding site, as indicated. On this probe, the HX-mutated form of Hoxb8 is able to form trimeric complexes with Pbx1/Meis1. An anti-HA against the HA tag of Hoxb8 was added in the last reaction to confirm the presence of trimeric complexes. Note that Pbx1 is able to bind the two TGAT binding sites of *PRS* (gray arrowheads). For all gels, colored bars and marks are symbolized as in Figure 3. See also Figure S9.

The contribution of the HX for Pbx1recruitment was analyzed by EMSAs in the mouse Hoxa1, Hoxb6, Hoxb7, Hoxb8, Hoxa9, and Hoxa10 proteins. Distinct DNA target sequences were used for anterior/central and posterior Hox proteins. The first nucleotide probe (called *ant/cent*; Figure 4A) contains sequences previously described as allowing Hox proteins from paralog groups 1 to 8 to form cooperative DNA-binding complexes with Pbx1 [12]. For posterior Hoxa9 and Hoxa10 proteins, one nucleotide of the *ant/cent* Hox/Pbx binding site (underlined in Figure 4A′ and [5]) was changed for converting the Hox core sequence to a consensus binding site for posterior Hox proteins (the resulting probe is called *post*). For both *ant/cent* and *post* probes, an identical core binding site for Meis1 (corresponding to the TGACAG consensus sequence; [6]) was added 8 nucleotides upstream, and in the same orientation, of the Hox/Pbx binding site. This topology was designed to mimic the regulatory element of Hoxa2, for which the contribution of the Meis binding site has been molecularly and functionally validated [10],[47]. On these probes, we found that the HX mutation either abolishes (for Hoxa1, Hoxb7, and Hoxa10; Figure 4A–A′) or strongly affects (for Hoxb6, Hoxb8, and Hoxa9; Figure 4A–A′) dimeric complex formation with Pbx1. In all but Hoxa1, the addition of Meis1 either completely (for Hoxb7, Hoxb8, Hoxa9, and Hoxa10) or partially (for Hoxb6) recues complex formation (Figure 4A–A′). These observations suggest that the Meis partner helps to promote HX-independent interaction modes, as previously observed with *Drosophila* Hox proteins. Moreover, the role of Meis1 is likely occurring through the remodeling of Hox-Pbx1 interactions since no Hox-Meis complexes are formed on the *ant/cent* or *post* probes, with wild type or HX-mutated Hox proteins (Figure S7).

We next investigated whether the contribution of Meis1 on Hox-Pbx1 interactions could depend on the nature of its binding site. This question was raised by the observation that the topology of the Meis binding site can strongly differ when comparing different characterized target enhancers, with strong variations in sequence, orientation, and distance from the Hox binding site (Figure S8 and [48]). Here we have tested whether Meis1 could still influence Hox-Pbx1 interactions when its binding site mimics the topology of the *Dllcon* and *Dll* repressor (*DllR*) elements [45]. To this aim, the Meis binding site of *ant/cent* and *post* probes was changed in one nucleotide position and reversed, leading to the *ant/cent^bis^* and *post^bis^* nucleotide probes (Figure 4B–B′). We observed that the HX mutation led to similar effects in the context of dimeric Hox-Pbx1 complexes on these modified nucleotide probes (Figure 4B–B′). The addition of Meis1 also led to a rescue of complex formation for Hoxb8, Hoxa9, and Hoxa10 (Figure 4B–B′). Surprisingly, the presence of Meis1 did not rescue complex formation for Hoxa7 (Figure 4B) and even provided constraints for restricting the complex assembly to a HX-dependent interaction mode in the case of Hoxb6 (Figure 4B).

Altogether, these in vitro experiments demonstrate HX dispensability for five of the six investigated mouse Hox proteins. Results also highlight a role for Meis1 in exerting a control over Hox-PBC interactions, as previously noticed with *Drosophila* proteins. This control appears to be influenced by the topology of the Hox/PBC/Meis binding sites in some instances.

### Meis DNA-Binding Is Important for Promoting HX-Independent Interactions between Hox and PBC Proteins In Vitro

The influence of the topology of the Hox/PBC/Meis binding sites suggest that binding of Meis proteins to DNA is a prerequisite for Meis-mediated uncovering of alternative Hox-PBC interaction modes. To investigate this more directly, we repeated EMSAs with DNA binding deficient Meis proteins. In the case of *Drosophila* proteins, we used a naturally HD-less isoform of Hth (Figure 5A), which contains only the evolutionary conserved HM domain mediating the direct interaction with Exd [49]. EMSAs were performed on *Dllcon*, which allowed assessing Hth-mediated uncovering of HX dispensability for Exd recruitment by Lab, Scr, Antp, and AbdB. The role of Hth-HM was not analyzed for Ubx and AbdA since these two proteins do not require Hth for establishing HX-independent interactions with Exd (Figure 3A). We observed that the HD-less isoform of Hth is able to form trimeric complexes in the context of wild type Hox proteins (Figure 5B). The HX mutation, however, abolishes the formation of the trimeric complex in all cases (Figure 5B), highlighting that Hth is not able to promote HX-independent interaction modes when it is not binding to *Dllcon* sequences.

EMSAs were also performed with mouse Hox proteins of central paralog groups, for which the HX dispensability for PBC recruitment was better (Hoxb6 and Hoxb8) or only (Hoxb7) uncovered in the presence of Meis1 on the *ant/cent* probe (Figure 4A). In this case, we used a full-length Meis1 protein mutated in the Asn54 of the HD. This mutant protein was called Meis^51^ (as it was done for Exd and Pbx1) since this residue corresponds to the position 51 in classical 60 amino acids long HDs [50]. Although this mutation abolished the DNA binding of Pbx1/Meis1 complexes (red asterisk in Figure 5C), as previously described [48], trimeric complexes were still observed with wild type Hox proteins (Figure 5C). These complexes are, however, lost in the context of the HX mutation (Figure 5C), highlighting that DNA binding deficient Meis1 is not able to uncover HX dispensability for Hox-Pbx1 interactions.

The importance of Meis DNA binding for unmasking HX-independent interaction modes between Hox and PBC proteins was further supported on *PRS* sequences. These sequences do not contain a Meis binding site and were originally described to promote HX-dependent interactions between several vertebrate Hox proteins and the Pbx1 cofactor [20],[22],[24]. Accordingly, we observed that the HX-mutated form of Hoxb8 cannot interact with Pbx1 on *PRS*, even in the presence of Meis1 (Figure 5D). In this last context, the simple addition of a Meis binding site is sufficient to partially restore the complex formation (Figure 5E), highlighting the importance of Meis DNA binding for promoting HX-independent Hox-Pbx1 interaction modes. Consistently, we observed that Meis1 is not able to promote HX-independent interaction modes between Hoxa9 or Hoxa10 and Pbx1 on the *post* probe lacking the Meis binding site (Figure S9).

### Conserved HX-Dispensability and Role of Meis Class Proteins in Controlling Hox-PBC Interactions in Cnidarians

The HX is widely dispensable for PBC recruitment in several *Drosophila* and mouse Hox proteins, suggesting that this property could be an ancestral character. To test this hypothesis we used two Hox proteins of the sea anemone *Nematostella vectensis*, which belongs to Cnidaria, the sister phylum of Bilateria.

**Figure 6:**
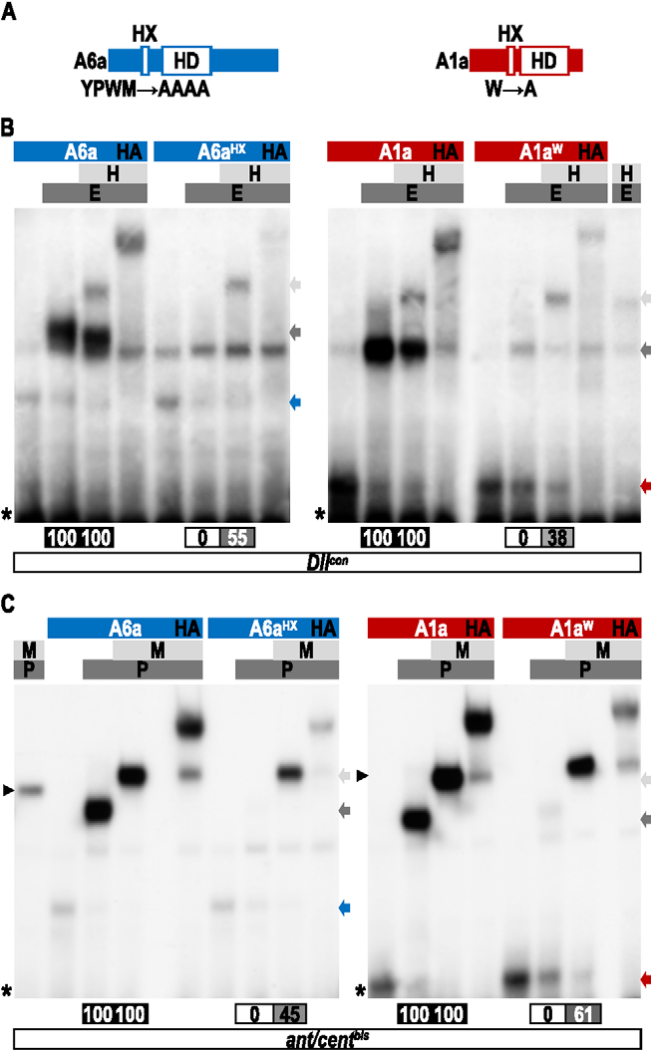
Hox proteins of the anthozoa *Nematostella vectensis* can recruit the PBC/Meis cofactors in absence of the HX in vitro. (A) Scheme of the Anthox6a and Anthox1a proteins. The HX mutation is indicated below each protein. (B) EMSAs of wild type or HX mutated forms of Anthox6a and Anthox1a with the *Drosophila* Exd or Exd/Hth cofactors on the *Dllcon* probe. (C) EMSAs with wild type or HX mutated forms of Anthox6a and Anthox1a with the mouse Pbx1 or Pbx1/Meis1 cofactors on the vertebrate *ant/cent^bis^* probe. In each condition, an anti-HA raised against the HA tag of Anthox proteins was added to verify the presence of trimeric complexes. Colored bars and marks are symbolized as in Figure 3. See also Figure S10.

Although Hox-like patterning functions remain hypothetical in this species [51],[52], Anthox6a or Anthox1a of *Nematostella* are, respectively, considered as anterior and central/posterior Hox proteins [51],[53],[54]. The interaction potential of Anthox6a or Anthox1a (Figure 6A) was analyzed by EMSAs with the mouse and *Drosophila* PBC/Meis cofactors. Results showed that Anthox6a and Anthox1a are able to form dimeric and trimeric complexes with Exd and Exd/Hth on *Dllcon*, respectively (Figure 6B). The HX mutation leads to a loss of dimeric complexes, while trimeric complexes are still present (with a mean loss of 50%; Figure 6B). Thus, the HX of Anthox proteins is not the unique Exd-recruiting domain in the presence of Hth.

Similar results were obtained with Pbx1 and Meis1 on the *ant/cent^bis^* probe (Figure 6C), again suggesting that Meis-class proteins are sufficient to promote HX-independent interaction modes in the assembly of Anthox/PBC/Meis complexes. Of note, no dimeric Anthox/Meis1 complexes are formed with wild type or HX-mutated proteins (Figure S10).

Although it remains to be determined whether HX-independent interactions could also exist in the context of PBC and Meis cofactors of *Nematostella vectensis*, our observations suggest that the potential for diversifying the PBC interaction mode already existed in Hox proteins of the last common ancestor of cnidarians and bilaterians.

### Revealing the Existence of Other Widely Used PBC Interaction Motifs in Two *Drosophila* Hox Proteins

The existence of HX-independent interaction modes between Hox and PBC proteins suggest that Hox proteins could contain other PBC interaction motifs. Besides the HX, only one other motif has been described to be necessary for PBC recruitment. This motif, called UbdA, is conserved among Ubx and AbdA proteins of protostomes [55] and was shown to mediate Ubx/Exd/Hth complex assembly on *cis*-regulatory sequences of the *Dll* target gene [28]. In addition, AbdA harbors an HX-like motif, TDWM, lying in between the canonical HX motif and the HD, and being evolutionary conserved in insect lineages [56].

**Figure 7:**
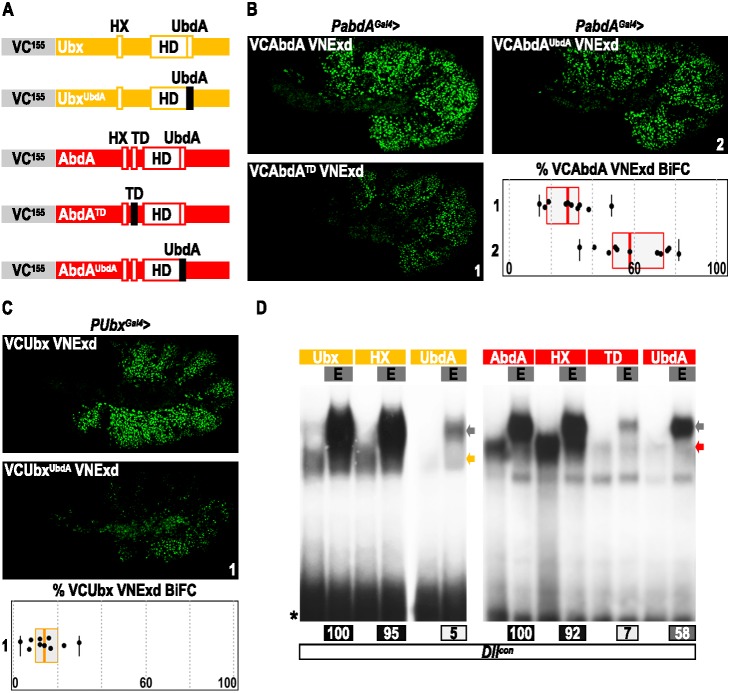
The *Drosophila* Ubx and AbdA proteins contain specific alternative PBC interaction domains. (A) Scheme of wild type and mutated VC-Ubx or VC-AbdA fusion proteins used for BiFC analysis with VN-Exd in the *Drosophila* embryo. (B) BiFC between Exd and the wild type, TDWM-mutated, or UbdA-mutated forms of AbdA, as indicated. Illustrative confocal captures of stage 10 living embryos are shown. Statistical quantifications of BiFC signals with the TDWM (1) or UbdA (2) mutation are depicted as a percentage of BiFC normally obtained with wild type AbdA. (C) BiFC between Exd and the wild type or UbdA-mutated form of Ubx, as indicated. Illustrative confocal captures of stage 10 living embryos are shown. Statistical quantifications of BiFC signals with the UbdA mutation (1) are depicted as a percentage of BiFC normally obtained with wild type Ubx. (D) EMSAs with wild type or mutated AbdA and Ubx proteins on *Dllcon*, as indicated. The TDWM and UbdA mutations drastically abolish AbdA/Exd and Ubx/Exd complex formation, respectively. Colored arrows indicate monomers and dimeric complexes as in Figure 3. See also Figure S11.

The global requirement of the TDWM and UbdA motifs for Exd recruitment in vivo was assessed by BiFC with the corresponding mutated VC-AbdA or VC-Ubx fusion proteins (Figure 7A). We observed that the TDWM or UbdA mutation in VC-AbdA leads to a mean loss of 60% and 50% of BiFC with Exd, respectively (Figure 7B) and that the UbdA mutation in VC-Ubx leads to a mean loss of 85% of fluorescence (Figure 7C). These distinct effects do not reflect differences in expression levels (Figure S11). The role of the TDWM and UbdA motifs as potential PBC-interacting motifs was also tested by EMSAs on *Dllcon*, which allows assessing the PBC-recruiting functions in the presence of Exd only. In contrast to the HX mutation, which has no effect, the TDWM and, to a lesser extent, UbdA mutations affect the AbdA/Exd complex formation (Figure 7D). Similarly, the UbdA mutation also strongly affects the Ubx/Exd complex assembly on *Dllcon* (Figure 7D).

Finally, the TDWM and UbdA motifs were also shown to be required for AbdA/Exd/Hth complex assembly on *DllR* sequences (Figure S12), confirming their importance in the context of physiological target sequences [56].We conclude that Ubx and AbdA, respectively, contain one (the UbdA motif) or two (the UbdA and TDWM motifs) other protein domains widely used for recruiting Exd.

## Discussion

### BiFC Reveals a Wide Dispensability of the HX Motif for Hox-PBC Interactions in Many Hox Proteins

Hox proteins reach functional specificity in part through their interaction with the common PBC class cofactors. The recruitment of the PBC cofactor is thought to rely on a unique HX-dependent association mode shared by all Hox proteins. This paradigm comes from the in vitro dissection of Hox/PBC complex assembly on a few specific DNA-binding sites, as well as from in vivo studies deciphering the molecular requirement for proper regulation of a few Hox/PBC target genes [28],[57]. While these approaches unambiguously establish a role for the HX in mediating Hox-PBC interactions, they do not question if other interaction modes exist and how widely these alternative modes contribute to Hox-PBC interactions. Here, our approach was distinct, using BiFC to address from a genome-wide perspective the contribution of the HX motif to Hox-PBC interactions in vivo.

Results obtained in cultured cells as well as in the developing *Drosophila* and chick embryos demonstrate that in the case of a few Hox proteins (3 out of the 12) the HX is predominant for mediating Hox-PBC interactions. The large majority of investigated Hox proteins are, however, not significantly impaired in their PBC interaction potential following HX mutation. These findings indicate that the HX is often and widely dispensable for Hox/PBC assembly in vivo.

A recent functional study of AbdA protein motifs corroborates our BiFC observations. In this work, the requirement of the HX has been assessed for a large set of AbdA-controlled events, including target gene regulation, phenotypic, and behavioral traits [56]. It was shown that the HX mutation was not essential for most Exd-dependent functions of AbdA, which could be explained by the takeover of the Exd-recruiting function by the alternative TDWM and UbdA motifs.

Importantly, our results do not contradict previous studies highlighting a role of the HX for Hox-PBC assembly, even for proteins for which we report a wide HX-dispensability. Our conclusions are drawn from the quantification of fluorescent signals in the entire nucleus, scoring Hox-PBC interactions at the genome-wide level, which represents hundreds of target loci. Through this approach, loss of HX-dependent Hox-PBC interactions at a few target loci would not be detectable. The absence of BiFC variations upon the HX mutation can thus not be interpreted as a complete dispensability for PBC recruitment, which is best illustrated by Antp that requires the HX to activate the *tsh* target gene [28], whereas BiFC reveals a wide dispensability. In summary our data indicate that the HX should not be considered as the unique and most frequently used Hox protein motif for PBC recruitment in vivo.

### Influence of the Meis-Class Proteins on the Mode of Hox-PBC Interactions

**Figure 8:**
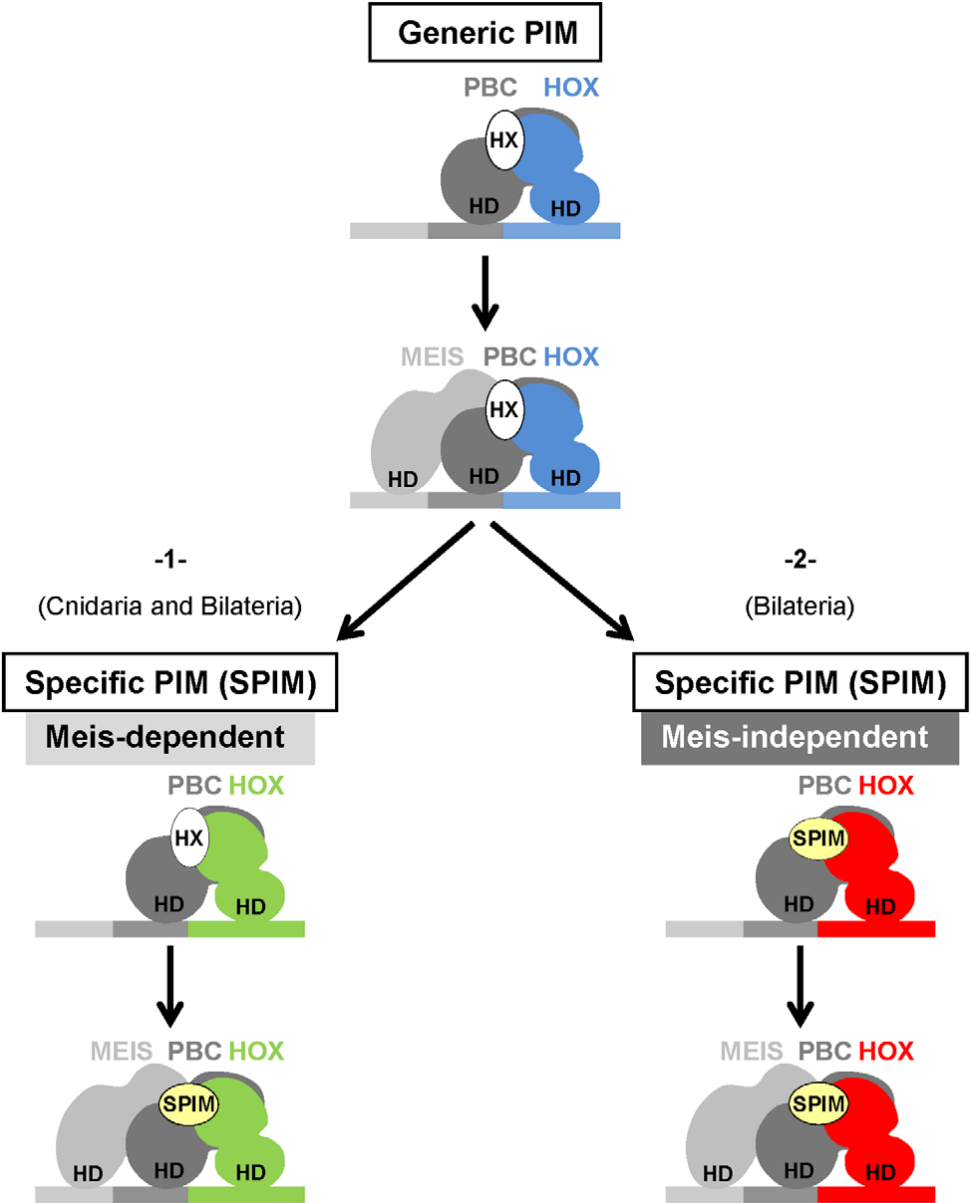
Role of Meis-class proteins in revealing alternative Hox-PBC interaction modes. In that model, the ancestral Hox-PBC interaction mode is thought to rely on the hexapeptide (HX), which we referred to here as a generic PBC interaction motif (PIM). The additional presence of Meis could have released some constraints, allowing Hox proteins to diversify their interaction mode with the PBC cofactor, as observed in cnidarian (Anthox6a and Anthox1a), *Drosophila* (Antp and AbdB), and mouse (Hoxb6, Hoxb7, Hoxb8, Hoxa9, and Hoxa10) Hox proteins (1). In other cases, Hox-PBC interaction modes could have evolved such that they do not absolutely require the presence of Meis to be HX-independent, as observed in *Drosophila* (Ubx and AbdA) and to a less extent in mouse (Hoxb6, Hoxb8, and Hoxa9) Hox proteins (2). In all cases, alternative Hox-PBC interaction modes will depend on specific PIMs (SPIMs). These motifs can be paralog-specific and conserved at various evolutionary extents, as illustrated by the UbdA and TDWM motifs. SPIMs could specify Hox functions by modulating their DNA-binding properties and regulatory potential, as discussed in the text.

The BiFC approach presents the advantage of measuring the interaction potential in the context of all partners that could assist Hox/PBC complex assembly in vivo. Such partners may influence the mode of Hox-PBC interactions, eventually leading to a dispensability of the HX for Hox/PBC complex formation. This hypothesis was tested by investigating the role of Meis-class proteins, which have been described to participate in Hox/PBC complexes for the regulation of several target genes. Our analysis established two modes of HX dispensability, which are dependent or not on the presence of Meis-class proteins (Figure 8). Results obtained with cnidarian Hox proteins also suggest that alternative Hox-PBC interaction modes were ancestrally Meis-dependent. Given that PBC and Meis proteins are thought to be constitutive interacting partners during embryogenesis, we speculate that the role of Meis on Hox-PBC interactions could potentially take place in most PBC-expressing cells. Thus, Hox proteins can be distinguished both by the nature of their PBC interaction mode and by their dependency towards the Meis partner for revealing it. The dependency towards Meis proteins was further shown to rely on Meis binding to DNA, as demonstrated by the strict requirement of Meis DNA-binding for unmasking HX-dispensability.

In *Drosophila*, it is interesting to note that the only Hox proteins which were described to achieve their regulatory activities in absence of HD-containing isoforms of Hth were Lab and Scr [49]. Accordingly, the formation of Lab/Exd/Hth or Scr/Exd/Hth complexes in vitro is more sensitive to the DNA-binding of Exd than to the DNA-binding of Hth [48]. On the contrary, Ubx and AbdA are more sensitive to the loss of Hth DNA-binding for trimeric complex assembly in vitro [48],[49] and for regulating their respective physiological target enhancers in vivo [49],[58]. These data suggest that Meis DNA-binding could be more critical for Hox proteins displaying alternative PBC interaction modes than for Hox proteins displaying a unique HX-dependent interaction mode.

The understanding of the molecular mechanisms by which Meis proteins could influence Hox-PBC interactions will require the resolution of Hox/PBC/Meis/DNA structures. We speculate that Hox-PBC interactions that are strongly remodeled by Meis likely rely on PBC-Meis and Hox-Meis interactions. Although the formation of PBC/Meis complexes is well established, interactions between Hox and Meis proteins were rarely described. Meis proteins can form cooperative DNA-binding complexes with vertebrate Hox proteins of posterior paralog groups [59], but interactions with more anterior Hox proteins have only been described in a DNA-binding-independent context [60]. In our EMSA experiments, no Hox-Meis DNA-binding complex was observed, except with the HX-mutated form of AbdB. Hox-Meis interactions could thus require the presence of the PBC cofactor to be stabilized, eventually leading to alternative Hox-PBC contacts. In that “ménage-à-trois,” the existence of Hox-PBC and Hox-Meis interactions have the advantage to expand the range of molecular strategies that could be used by Hox proteins to assemble into a trimeric complex.

In the case of Hoxb6, results highlighted a different role for the Meis partner, restricting the interaction potential to an HX-mediated interaction on the *ant/cent^bis^* probe. Meis-class proteins can thus exert two opposite roles on Hox/PBC assembly. Both roles are consistent with a function influencing the conformation of the Hox and/or PBC proteins, which might ultimately result either in favoring HX-alternative modes of PBC interaction or in restricting it to an HX-dependent mode. The output of Meis-mediated changes in Hox and/or PBC conformations depends on the identity of the Hox protein, but also on specific features of the DNA target sequence, as evidenced by the observation that binding sites can be completely biased towards HX-dependent modes of PBC recruitment (example of *PRS* for Hoxb8 or *ant/cent^bis^* for Hoxb7), even in the presence of the Meis partner (example of *ant/cent^bis^* for Hoxb6 and Hoxb7). Such allosteric effects of DNA on protein complex assembly have also been described in a few instances [15],[57],[61].

### Diversity in Hox-PBC Interaction Modes: Implications for Hox Protein Function

The dispensability of the HX for PBC recruitment in several central and posterior Hox proteins implies the existence of multiple Hox-PBC interaction modes, which was experimentally investigated in the case of the *Drosophila* Ubx and AbdA proteins. BiFC showed that the wide dispensability of the HX was due to the preponderant role of one motif in Ubx, the UbdA motif, or two motifs in AbdA, the TDWM and UbdA motifs. This use of protein motifs for Hox-PBC complex assembly at the genome-wide scale is also apparent at the level of a single Hox-PBC target gene, as exemplified by the regulation of the *DllR* enhancer (this study and [28],[56]). The PBC-recruiting motifs of AbdA were also described to act in a selective, additive, or interactive way for controlling other known PBC-dependent functions [56], illustrating the potential extreme modularity of PBC interaction modes depending on the Hox protein and/or the developmental context considered. Interestingly, the UbdA motif is only found in Ubx/AbdA paralog proteins of protostomes [55], while the TDWM motif is only found in AbdA proteins of insects [56], supporting the view that diversifying Hox-PBC interaction modes could set the bases for distinguishing Hox protein functions from different paralog groups.

Distinct Hox-PBC interaction modes have recently been proposed to affect the way Hox proteins interact with DNA. This model is supported by experimental evidence showing that paralog-specific motifs could facilitate the interaction with the PBC cofactor and hence the cooperative DNA-binding of the resulting dimeric complex [44]. In addition, it was also noticed that the regulation of specific Scr, Ubx, and AbdA target genes correlated with the use of different PBC-recruiting motifs for the complex assembly [62]. This result suggests that distinct Hox-PBC interaction modes could be responsible for distinct DNA-binding specificities in vivo. The underlying molecular mechanisms remain, however, difficult to apprehend, since no target enhancer has been shown to be regulated by a specific Hox-PBC interaction mode. On the contrary, the few paralog-specific target enhancers characterized so far often tolerated the DNA-binding of at least two different Hox/PBC/Meis complexes (see, for example, [57] or [48]).

Finally, Hox functional specificity has also been proposed to rely on mechanisms controlling the regulatory potential [26],[63],[64]. Within this functional context, distinct Hox-PBC interaction modes could influence post-DNA-binding regulatory mechanisms for specifying Hox transcriptional activities. This hypothesis is supported by a recent study showing that switching the Ubx/Exd/Hth complex assembly from an UbdA to a HX-dependent interaction mode decreased the overall repressive activity of the complex on the *Dll* enhancer without affecting its DNA-binding properties [65]. Thus, multiple Hox/PBC interaction modes could also play important roles for shaping the interface between Hox/PBC/Meis complexes and specific corepressors and/or coactivators. Such a molecular strategy could help discriminating Hox functions in absence of clear distinct DNA-binding preferences.

## Materials and Methods

### Fusion Protein Constructs and Transgenic Lines

Hox and Pbx variants were generated by PCR from full-length complementary DNAs and restriction-cloned in fusion with the C-terminal (VC) or N-terminal (VN) fragment of Venus in the appropriate vector (see Table S1 for a complete list of all constructs). Primers used are available upon request. For all fusion constructs, a linker of five amino acids was added to separate the Venus fragment from the protein of interest. All constructs were sequence-verified before using. Transgenic lines were established either by the PhiC-31 integrase system (with the pUASTaatB vector; [38]) or by classical P-element (with PUAST vector) mediated germ line transformation. Experimental conditions allowing physiological levels of expression were established as already described [37].

### Fly Stocks

Gal4 drivers used are: *engrailed (en)-Gal4*, *Antp-Gal4*, *Ubx-Gal4*, *abdA-Gal4*, and *AbdB-Gal4* [37],[39].

### BiFC Analysis in *Drosophila* Embryos

Fly crosses for BiFC analyses were set up at the defined temperature overnight. After removal of the flies, embryos were kept at 4°C for 28 h before live imaging. To visualize complementation between split Venus fragments, living embryos were dechorionated and mounted in the halocarbon oil 10S (commercialised by VWR). To quantify the BiFC signals, unsaturated images of ectodermal fluorescence were taken in embryos of desired stage (with a minimum of 10 embryos by condition) using a LSM510 confocal microscope (Zeiss). For Venus fluorescence, filters were adjusted at 500 nm for excitation and 535 nm for emission. Identical parameters of acquisition were applied between the different genotypes. Number and intensity of all the pixels (for each embryo) were measured using the histogram function of the ImageJ Software. Quantification of fluorescence complementation was shown for each condition by boxplot representation using R-Software. Boxplot depicts the smallest value, lower quartile, median, upper quartile, and largest value for each condition. Black points correspond to individual measures.

### BiFC Analysis in the Trunk Neural Tube of Chick Embryos

Fertilized chick eggs were obtained from a commercial source (EARL Morizeau, Dangers, France) and incubated at 38°C in a humidified incubator. Embryos were staged according to the developmental table of Hamburger and Hamilton “HH” (1992) or according to days of incubation. All electroporations were performed *in ovo*. Embryos were co-electroporated with a vector encoding a red fluorescent protein (pIRES-2-mCherry; 0.05 µg/µl) and two BiFC vectors (0.7 µg/µl each). The DNA was microinjected into the lumen of the neural tube at the trunk level of HH10–11 chick embryos with micropipettes. Platinum electrodes were placed parallel to the trunk neural tube and placed just beneath the surface, submerged in albumen. A square-wave stimulator (Intracell, TSS20 ovodyne electroporator, and EP21 current amplifier) was used to deliver five pulses of current at 25 volts for 50 ms wide in a 50 ms interval. With unilateral pulses, only the right part of neural tubes is transfected. Embryos were allowed to develop for 24 h and were processed for visualization. To quantify BiFC, unsaturated images of neural tube fluorescence were taken (with a minimum of 10 embryos by condition) using a LSM510 confocal microscope (Zeiss). Identical parameters of acquisition were applied between the different genotypes. Each experiment was conducted independently three times.

### BiFC Analysis in COS7 Cells

COS7 cells were maintained in Dulbecco's modified Eagle's medium (1 g/L glucose, Invitrogen) supplemented with10% foetal bovine serum (Invitrogen), 100 IU/ml penicillin, and 100 µg/ml streptomycin (Invitrogen) at 37°C in a humidified 5%CO_2_ atmosphere. 24 h before transfection, 10^5^ cells were plated on glass coverslips. Transfections were carried out using the JetPRIME reagent (Polyplus), with a total amount of 500 ng of DNA: 300 ng of the VN- fusion vector, 70 ng of the VC-fusion vector, and 130 ng of pCat. Cells were fixed 24 h after transfection, and coverslips were mounted on glass slides with Vectashiel+Dapi (Vector) and observed under a confocal microscope. For control experiments, fixed cells were submitted to an immmunohistochemistry using a rabbit polyclonal anti-GFP (Invitrogen A11122) diluted 1/200 as primary antibody, and a goat anti-rabbit IgG-AF555 (Molecular Probes 4413) diluted 1/750 as secondary antibody.

### Immunostainings

Immunodetections in *Drosophila* embryos, chick embryos, and COS7 cells were performed according to standard procedures. The antibodies used were: rat anti-HA (Molecular probe, Invitrogen, CA, USA; 1/500), chicken anti-GFP (Promega, WI, USA; 1/500 in *Drosophila* and chick embryos), and rabbit anti-GFP (Invitrogen A11122; 1/200 in COS7 cells).

### Protein Expression and Electrophoretic Mobility Shift Assays

Constructs for EMSAs were cloned in the PcDNA3 vector and sequence-verified before using. Proteins were produced with the TNT-T7-coupled in vitro transcription/translation system (Promega). Production yields of wild type and mutated counterpart proteins were estimated by ^35^S-methionine labeling. EMSAs were performed as described previously. The sequence of the *Drosophila* labeled probes is provided below: *DllR^con^* : 5′-TATTTGGGCCATAAATCATTCCCGCGGACAGTT-3′, *EVIII* : 5′-TTTGTCGCAATCGTGATCAATTACAGCTGACTGGGTTG-3′, *apME680* site 1: 5′-TGAAATGCGCCAATTATTTTGATTAATGCCAAAGAA-3′; Box2 from the *tsh* epidermal enhancer: 5′-TCATGGACTGAAAACCATAAATTTGATAATTGACTTTCCAC-3′, *Rho* : 5′-CAGTTCATTGATTGACATTTTTATTATGCATATTC-3′, *CycE* : 5′-TCTGATCAATGTCAAAAGATAATTTATTATTTGAGTAGCCTTTAA-3′, *Fkh* : 5′- CTCAATGCAAGATTAATCGCCAGCTGTGGGACGAGG-3′; *DllR*: 5′-TATTTGGGAAATTAAATCATTCCCGCGGACAGTT-3′.

## Supporting Information

Figure S1 Quantification of the expression level of wild type and HX-mutated Hox fusion proteins in the *Drosophila* embryo. Expression levels were assessed with an anti-GFP antibody recognizing the C-terminal part of Venus (see also Materials and Methods). Boxplots on the right show the statistical quantification of the expression level of the mutated Hox fusion protein (numbers) with regard to the expression level of the corresponding wild type fusion protein.
(TIF)

Figure S2 Absence of BiFC signals between the mesoderm-specific transcription factor Collier (Col) and the *Drosophila* Antp, Ubx, AbdA, or AbdB proteins. Fusion proteins were specifically expressed in the somatic mesoderm with the *24B-Gal4* driver. (A) Confocal pictures of stage 14 embryos expressing the VN-Col and VC-Hox fusion proteins, as indicated. In all cases, no BiFC signals are visible, suggesting that Col is not a direct cofactor of Hox proteins in the mesoderm. (B) Expressing VC-AbdA and VN-Exd with *24B-Gal4* confirms that the somatic mesoderm is a suitable tissue for BiFC. (C) Control experiment showing that the fusion proteins are correctly expressed in the somatic mesoderm, as attested by the anti-Col (red) and anti-GFP (recognizing the VC and VN fragments of fusion proteins, white) immunostainings.
(EPS)

Figure S3 *Drosophila* Hox proteins do no form dimeric complexes with the HD^51^-mutated form of Exd in vitro. (A) The HD^51^ mutation abolishes monomer DNA-binding of Exd on the *PRS* nucleotide probe. (B) The HD^51^ mutation of Exd abolishes dimeric complex formation with Lab, Scr, Antp, and Ubx on the *Dll^con^* probe. Control experiments were performed with wild type Exd. Colored bars and marks are symbolized as in Figure 3.
(EPS)

Figure S4 In vivo interactions between HX-mutated VC-Antp or VC-AbdB and VN-Exd occur on DNA. The illustrative confocal capture is shown for a stage 10 embryo. The statistical quantification of BiFC signals is shown on the right as a boxplot. BiFC with the HD-mutated form of Exd is indicated as a percentage of the BiFC normally obtained with wild type Exd.
(EPS)

Figure S5 Wild type and mutated fusion constructs are similarly expressed in COS7 cells or in the trunk neural tube of chick embryos. (A) Expression of wild type or HX and HD-mutated forms of Hox and Pbx1 fusion proteins in COS7 cells, respectively. Nuclei are stained by DAPI (blue) and fusion constructs are revealed with a polyclonal anti-GFP recognizing the VC and VN fragments (orange). (B) Expression of the wild type and HD-mutated form of Pbx1 in the trunk neural tube of chick embryos. Nuclei are stained by DAPI (blue) and fusion proteins by the polyclonal anti-GFP (green). Immunostaining was performed on 18 µm cryostat transversal sections.
(TIF)

Figure S6 *Drosophila* Hox proteins do not form DNA-binding complexes with Hth in vitro. (A) EMSAs with wild type *Drosophila* Hox proteins and Hth (H) on the *Dll^con^* nucleotide probe, as indicated. No dimeric complex is formed. Note that Lab and Scr are not able to bind as a monomer on *Dll^con^*. (B) EMSAs with HX-mutated *Drosophila* Hox proteins and Hth on the *Dll^con^* nucleotide probe, as indicated. No dimeric complex is formed, except with AbdB (gray arrow). Note that the HX mutation allows Lab to bind DNA, as previously described on another probe [33], and increases the monomer binding activity of Antp. Black arrowhead indicates nonspecific binding of lysat (L) products. (C) Exd interacts with the HX-mutated form of AbdB on *Dll^con^*. The presence of Exd in the trimeric complex was validated by a supershift with a polyclonal anti-Exd antibody (last lane). Note that the trimeric complex migrates more rapidly than the dimeric AbdB/Hth complex. (D) Exd interacts with the HX-mutated form of AbdB on the *CycE* probe. The presence of Exd in the trimeric complex was validated by a supershift with a polyclonal anti-Exd antibody (last lane). On this probe, AbdB/Hth complexes are hardly seen under longer exposition times (not shown). Colored bars and marks are symbolized as in Figure 3.
(EPS)

Figure S7 Mouse Hox proteins do no form dimeric complexes with Meis1 in vitro. (A) EMSAs between wild type or HX-mutated Hox proteins of central paralog groups and Meis1 on the *ant/cent* probe, as indicated. Black arrowhead indicates nonspecific binding of lysat (L) products. (A′) EMSAs between wild type or HX-mutated Hoxa10 and Meis1 on the *post* probe, as indicated. EMSAs were not performed with Hoxa9 since the HX-mutated form of this protein is able to strongly interact with Pbx1 in absence of Meis1 (Figure 4A′). Colored bars and marks are symbolized as in Figure 3.
(EPS)

Figure S8 Sequence and orientation of Hox, Exd, and Hth binding sites in characterized *Drosophila* enhancers.
(EPS)

Figure S9 HX-mutated Hox proteins of posterior paralog groups cannot form trimeric complexes with Pbx1 and Meis1 on the *post* probe in absence of Meis binding sites. (A) EMSA between wild type or HX-mutated Hoxa9 and Pbx1 or Pbx1/Meis1 on the *post* probe lacking the Meis binding site, as indicated. (B) EMSA between wild type or HX-mutated Hoxa10 and Pbx1 or Pbx1/Meis1 on the *post* probe lacking the Meis binding site, as indicated. Red asterisk indicates absence of dimeric Pbx1/Meis1 complexes normally formed on the *post* probe (Figure 4B′). Colored bars and marks are symbolized as in Figure 3.
(EPS)

Figure S10 Hox proteins of the anthozoa *Nematostella vectensis* cannot form dimeric complexes with Meis1. EMSAs were performed with wild type or HX-mutated forms of Anthox proteins and Meis1 on the *ant/cent^bis^* probe, as indicated. Black arrowheads indicate nonspecific binding of lysat (L) products. Colored bars and marks are symbolized as in Figure 3.
(EPS)

Figure S11 Quantification of the expression level of wild type and mutated Ubx and AbdA fusion proteins in the *Drosophila* embryo. Expression levels were assessed as in Figure S1.
(EPS)

Figure S12 Role of the HX, TD, and UbdA motifs of AbdA for complex formation with Exd/Hth on *DllR* sequences. Colored bars and marks are symbolized as in Figure 3.
(EPS)

Table S1 Cloning strategies of Hox, PBC, and Meis constructs used in this study. The table indicates: fusion topologies with Venus fragments, nature of the mutations engineered, restriction sites used for cloning, and vectors and genomic insertion sites used for establishing *Drosophila* fly lines.
(XLS)

## Abbreviations

AbdA: AbdominalA
AbdB: AbdominalB
Antp: Antennapedia
AP: anterior-posterior
BiFC: Bimolecular Fluorescence Complementation
Col: Collier
Dfd: Deformed
*Dll*: *Distalless*
*DllR*: *Dll* repressor
EMSAs: electrophoretic mobility shift assays
*en*: *engrailed*
Exd: Extradenticle
HD: homeodomain
*hth*: *homothorax*
HX: hexapeptide
Lab: Labial
*Scr*: *Sex combs reduced*
Ubx: Ultrabithorax
VC: C-terminal part of Venus
VN: N-terminal part of Venus
