# Supplementary figures and images for: Hox Proteins Display a Common and Ancestral Ability to Diversify Their Interaction Mode with the PBC Class Cofactors
Source: PLoS Biol. 2012 Jun 26;10(6):e1001351. doi: 10.1371/journal.pbio.1001351 (PMC3383740; doi:10.1371/journal.pbio.1001351)

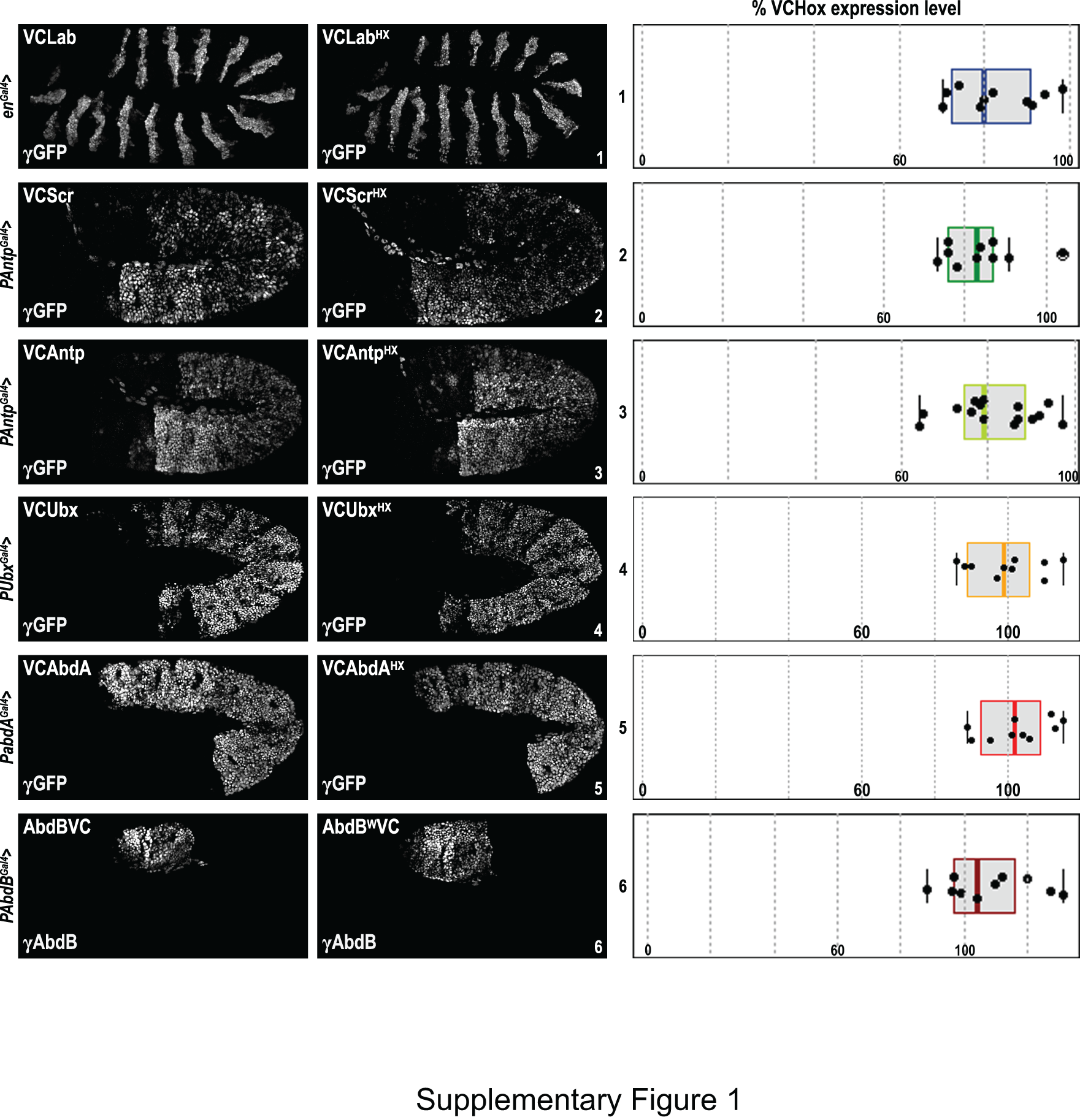

Supplement: Figure S1 — Quantification of the expression level of wild type and HX-mutated Hox fusion proteins in the Drosophila embryo. Expression levels were assessed with an anti-GFP antibody recognizing the C-terminal part of Venus (see also Materials and Methods). Boxplots on the right show the statistical quantification of the expression level of the mutated Hox fusion protein (numbers) with regard to the expression level of the corresponding wild type fusion protein. (TIF) [file pbio.1001351.s001.tif]

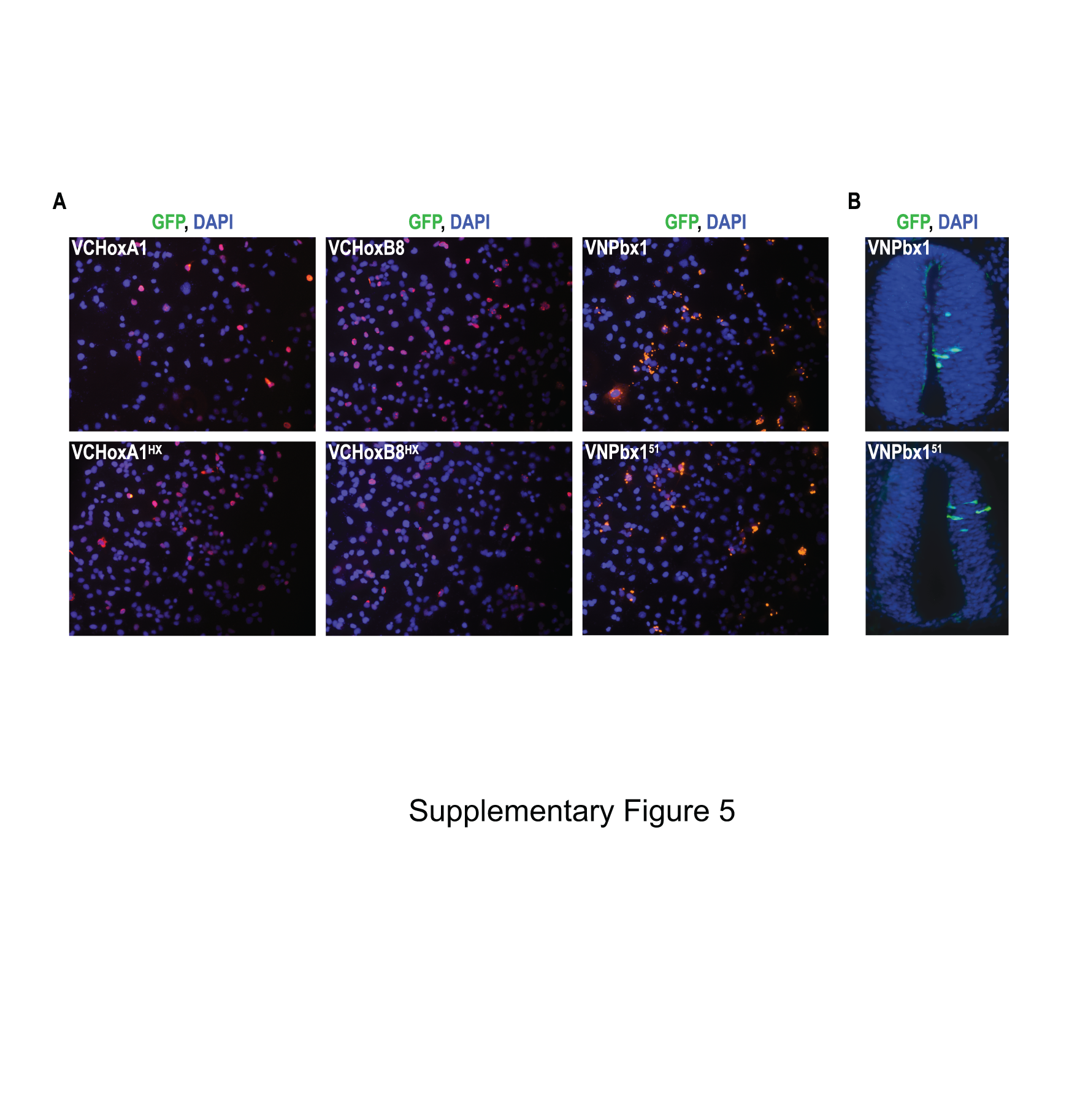

Supplement: Figure S5 — Wild type and mutated fusion constructs are similarly expressed in COS7 cells or in the trunk neural tube of chick embryos. (A) Expression of wild type or HX and HD-mutated forms of Hox and Pbx1 fusion proteins in COS7 cells, respectively. Nuclei are stained by DAPI (blue) and fusion constructs are revealed with a polyclonal anti-GFP recognizing the VC and VN fragments (orange). (B) Expression of the wild type and HD-mutated form of Pbx1 in the trunk neural tube of chick embryos. Nuclei are stained by DAPI (blue) and fusion proteins by the polyclonal anti-GFP (green). Immunostaining was performed on 18 µm cryostat transversal sections. (TIF) [file pbio.1001351.s005.tif]
